# Perceptions and Expectations of Pharmacist Interventions in Adverse Event Management During Drug Therapy for Metastatic Renal Cell Carcinoma: A Cross-Sectional Survey in Japan

**DOI:** 10.3390/cancers17243951

**Published:** 2025-12-11

**Authors:** Tetsuya Wako, Go Kimura, Yasuhisa Fujii, Takahiro Osawa, Yosuke Uchitomi, Kazunori Honda, Miki Kondo, Ariko Otani, Yoshihide Mitsuda, Nobuo Shinohara

**Affiliations:** 1 Department of Pharmacy, Nippon Medical School Musashikosugi Hospital, Kawasaki 211-8533, Japan; 2 Department of Urology, Nippon Medical School Hospital, Tokyo 113-8603, Japan; 3 Department of Urology, Institute of Science Tokyo, Tokyo 113-8510, Japan; 4 Department of Urology, Hokkaido University Hospital, Sapporo 060-8638, Japan; 5 Department of Cancer Survivorship and Digital Medicine, The Jikei University School of Medicine, Tokyo 105-8461, Japan; 6 Department of Clinical Oncology, Aichi Cancer Center, Nagoya 464-8681, Japan; 7 Department of Nursing, National Cancer Center Hospital East, Kashiwa 277-8577, Japan; 8 Eisai Co., Ltd., Tokyo 112-8088, Japan; 9 Department of Urology, Kushiro Rosai Hospital, Kushiro 085-0052, Japan

**Keywords:** adverse event, cross-sectional survey, multidisciplinary management, perceptions, pharmacist, renal cell carcinoma

## Abstract

**Background:** We investigated the role of pharmacists in adverse event (AE) management during renal cell carcinoma (RCC) drug therapy by surveying patients, physicians, and pharmacists. We identified the types of AEs for which pharmacist involvement is beneficial and explored measures to promote pharmacist intervention. **Methods:** This was an ad hoc analysis of a questionnaire-based cross-sectional web survey conducted from May to June 2022 among patients undergoing RCC drug therapy, physicians prescribing RCC treatments, and pharmacists involved in oncology care in Japan. **Results:** A total of 83 patients with metastatic RCC, 165 physicians, and 218 pharmacists were included. Among patients, 28.9% reported experiencing AEs or symptoms requiring pharmacist intervention. Most physicians (78.2%) and pharmacists (96.3%) supported pharmacist involvement in AE management. Notably, 35.6% of patients who reported no AEs or symptoms requiring pharmacist intervention acknowledged difficulty in communicating AEs to their physicians. Regarding desired pharmacist interventions for AEs, patients prioritized rash/pruritus, fatigue, and diarrhea; physicians emphasized stomatitis and anorexia; pharmacists identified constipation, stomatitis, and diarrhea. The most common reason patients valued pharmacist involvement was the reassurance of support from multiple healthcare providers. Physicians and pharmacists valued pharmacists’ greater familiarity with AE management, particularly considering physicians’ limited time. Raising awareness among patients and healthcare professionals, patient requests, and improving institutional support were strategies to enhance pharmacist involvement. Over 86% of healthcare professionals considered pharmaceutical outpatient clinics necessary to strengthen interdisciplinary collaboration. **Conclusions:** This study highlights widespread support among patients, physicians, and pharmacists for pharmacist involvement in managing AEs during RCC drug therapy.

## Simple Summary

Multidisciplinary management can be helpful for patients to manage side effects from cancer treatment. However, in Japan, the role of pharmacists in systemic therapy of renal cell carcinoma has not been clearly defined. In this study, we surveyed patients, doctors, and pharmacists to understand their opinions and expectations of the role pharmacists should take in helping patients manage side effects during treatment. We found that there is a gap between patients and healthcare professionals in their needs of pharmacist involvement, as well as the side effects that they consider require intervention. This suggests that a better understanding of the role of pharmacists in clinical practice may lead to improved side effect management. Furthermore, most healthcare professionals considered pharmaceutical outpatient clinics necessary to strengthen teamwork among different healthcare workers. Overall, we found that there are issues and potential benefits for pharmacist involvement in managing side effects during renal cell carcinoma treatment.

## 1. Introduction

Recently, multiple drug regimens have been used to treat renal cell carcinoma (RCC) in Japan, including five types of combined immunotherapy [1]. Introducing these regimens has dramatically improved treatment outcomes for patients with metastatic RCC (mRCC), contributing to longer survival and improved quality of life [2]. These improvements have been accompanied by an increase in the duration of treatment for many patients with mRCC, leading to an increase in the types and frequency of adverse events (AEs) associated with their medication, each requiring appropriate management.

In a previous study, we conducted a web-based questionnaire survey to identify the concerns of patients with mRCC during drug therapy and to understand physicians’ perceptions of those concerns [3]. For both groups, the most common concern was how daily activities were affected by AEs, highlighting the importance of managing AEs during RCC treatment. Furthermore, in the same study, 50.6% of patients said that they found it challenging to communicate their AEs to their physician, particularly AEs such as fatigue/malaise, anxiety, and depression. The main reasons given for finding communication difficult included that they thought they had to be patient and that they did not know how to tell physicians about their AEs.

Other studies have reported differences between patient-reported AEs and those reported in clinical trials during the treatment of mRCC [4]. In the FAMOUS study of RCC treatment with molecular-targeted drugs, patients’ and physicians’ perceptions of fatigue as an AE differed [5]. These findings suggest that physicians may not always be fully aware of the AEs that patients experience during treatment, making it harder to detect and manage them.

A multidisciplinary approach to managing AEs caused by anticancer drugs, in which patients are cared for by a team of professionals rather than physicians only, is a positive step in cancer care [6,7]. Teams addressing cancer drug therapy comprise diverse health and social care professionals. In particular, the involvement of pharmacists, whose duties include dispensing and managing medication, is becoming more important as the number of cancer patients being treated as outpatients, rather than inpatients, increases [8].

In 2006, Board-certified Oncology Pharmacist was established as a qualification specializing in chemotherapy in Japan, and the proportion of hospitals enrolling pharmacists with oncology-related certifications has since significantly increased [9]. Several reports suggest that pharmacist involvement in systemic cancer therapy can be useful because it facilitates the early detection of and response to AEs resulting from oral anticancer treatments such as sunitinib and sorafenib [10,11,12]. The usefulness of pharmacists is not limited to the management of oral anticancer drugs. Similar results have also been obtained for managing immune-related AEs arising from immune checkpoint inhibitor (ICI) treatment [13,14], with physicians expecting pharmacists to help manage immune-related AEs during such treatment [15]. Therefore, pharmacist intervention is thought to be important in managing AEs during cancer treatment and will likely bring various benefits to patients. However, it is unclear exactly what patients and physicians expect regarding pharmacist involvement, particularly when it comes to managing specific AEs during drug therapy or in determining how to promote pharmacist involvement. In this study, we surveyed patients, physicians, and pharmacists to identify expected pharmacist involvement in managing AEs, what AEs may benefit from being addressed by pharmacist involvement, reasons why pharmacist intervention was needed, and what is needed to promote pharmacist intervention during RCC drug therapy.

## 2. Materials and Methods

### 2.1. Study Design and Participants

This was an ad hoc analysis of a cross-sectional web survey study conducted during May and June 2022 in Japan. The detailed methods have been published previously [3,16]. The study population included patients aged ≥20 years with mRCC who underwent systemic therapy, including molecular targeted therapy or ICI therapy (combination therapy); who were living in Japan; and who agreed to participate in the web questionnaire survey (Supplementary Table S1). Eligible physicians included those providing care to patients with mRCC, who had prescribed systemic therapy for RCC, and who consented to participate in the web questionnaire survey. Eligible pharmacists consisted of those who agreed to take part in the web questionnaire survey and were involved in any systemic cancer therapy. The study excluded patients who were or were associated with healthcare professionals, pharmaceutical or marketing companies; patients with multiple cancers; physicians not prescribing RCC systemic therapy; and pharmacists not involved in systemic cancer therapy.

### 2.2. Outcomes

The primary outcomes were the percentage of RCC patients, physicians, and pharmacists who considered that pharmacist intervention was necessary for managing AEs; AEs associated with RCC drug therapy that patients, physicians, and pharmacists believed required pharmacist intervention; reasons why RCC patients, physicians, and pharmacists felt that pharmacist intervention was needed; and measures deemed necessary to enhance pharmacist involvement in the management of AEs.

### 2.3. Sample Size

The sample size of each target population was based on the number of patients and physicians registered in the web-based survey panels (95 patients, 150 physicians, and 180 pharmacists).

### 2.4. Ethical Considerations

All study procedures were conducted in accordance with the principles and guidelines outlined in the Declaration of Helsinki. The Research Ethics Committee of the Japanese Association for the Promotion of State-of-the-Art in Medicine approved the study protocol. All collected data were kept confidential and used solely for research purposes. Each participant provided written informed consent.

## 3. Results

### 3.1. Participant Characteristics

The results of the participant screening are shown in Supplementary Figure S1a–c. From May to June 2022, 101 patients with mRCC were screened; after excluding 6 for invalid answers and 12 based on exclusion criteria, 83 remained for analysis. Of 183 physicians screened, 18 were excluded due to invalid answers, leaving 165 for data analysis. In total, 221 pharmacists were screened for this study; three gave invalid responses and were excluded, leaving 218 included in the analysis.

The background characteristics of the participants are shown in Table 1. The mean ± standard deviation (SD) age of patients with mRCC was 44.2 ± 12.2 years, with 63.9% identifying as men. All patients received systemic treatment for mRCC, with 92.8% still undergoing this therapy. Systemic therapy duration varied, with 30.1% receiving it for <6 months, 34.9% for between 6 months and 2 years, and another 34.9% for >2 years. The mean ± SD career length for physicians was 18.1 ± 7.6 years. The settings where physicians mainly practiced included general hospitals (43.6%), public hospitals (32.1%), university hospitals (21.2%), and private clinics (3.0%). The most common physician specialty was urology, accounting for 62.4% of respondents, followed by medical oncology at 30.3% and renal transplant surgery at 7.3%. The mean ± SD career length as a pharmacist was 18.5 ± 8.4 years. The main work locations for pharmacists were pharmacies/drugstores (42.2%), general hospitals (29.8%), public hospitals (17.0%), university hospitals (10.1%), and private clinics (0.9%). In the previous year, 49.1% of the pharmacists surveyed provided drug therapy services for patients with RCC.

### 3.2. Patient, Physician, and Pharmacist Views on Pharmacist Involvement in RCC Treatment AE Management

**Figure 1:**
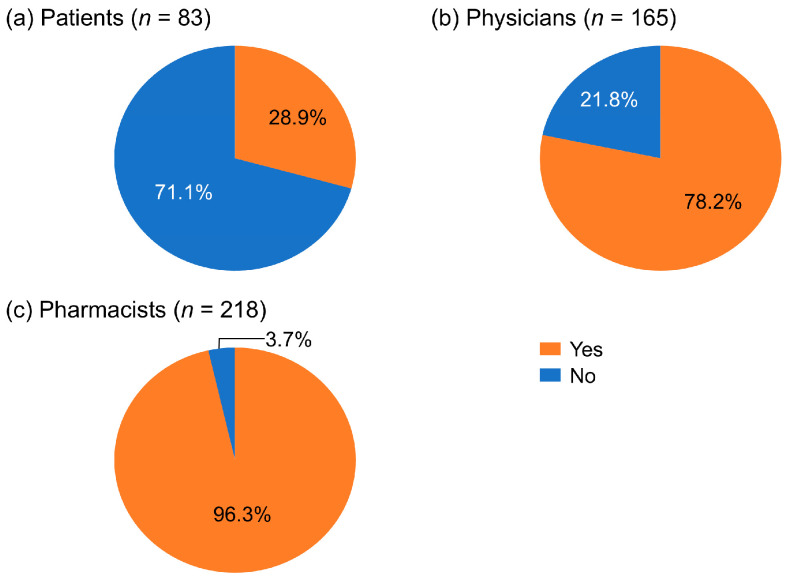
Percentages of patients with renal cell carcinoma, physicians, and pharmacists who considered pharmacist involvement necessary for managing adverse events.

In total, 28.9% of RCC patients reported they had AEs or symptoms that needed pharmacist intervention (Figure 1a). Of the physicians who responded, 78.2% thought pharmacists should be involved in managing AEs and symptoms (Figure 1b). Among the pharmacists surveyed, 96.3% responded that they felt that AE or symptom management required pharmacist intervention (Figure 1c). Of the 71.1% of RCC patients who indicated that they had “nothing in particular” regarding their AEs or symptoms requiring pharmacist intervention, 35.6% responded that they had AEs that were difficult to communicate to their physician.

### 3.3. AEs Associated with RCC Treatment Needing Pharmacist Intervention

**Figure 2:**
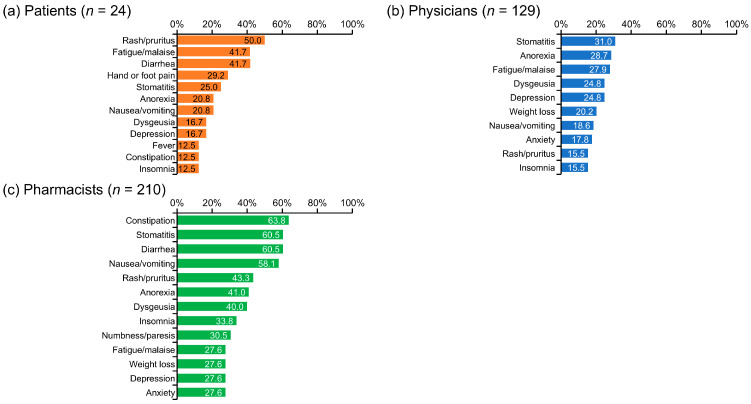
Adverse events or symptoms that required pharmacist intervention. This figure corresponds to the following survey questions: (**a**) Q25: Of the AEs and symptoms from drug therapy for renal cell carcinoma that you have experienced up to this time, are there any for which you feel support from a pharmacist is necessary? (Select all that apply) (**b**) Q25: Of the AEs and symptoms associated with drug therapy for renal cell carcinoma, are there any for which you feel interventions by pharmacists are necessary? (Select all that apply) (**c**) Q26: Of the AEs and symptoms associated with drug therapy for renal cell carcinoma, are there any for which you feel interventions by a pharmacist are necessary? (Select all that apply) AE, adverse event.

Rash/pruritus (50.0%), fatigue/malaise (41.7%), and diarrhea (41.7%) were the most frequently cited AEs or symptoms for which patients perceived a need for pharmacist intervention (Figure 2). Physicians most commonly identified the AEs of stomatitis (31.0%), anorexia (28.7%), and fatigue/malaise (27.9%) as needing pharmacist intervention. In contrast, pharmacists emphasized a broader range of symptoms, with constipation (63.8%), stomatitis (60.5%), diarrhea (60.5%), and nausea/vomiting (58.1%) standing out as the most frequent. Notably, there was some overlap in the most highly ranked AEs across groups, including stomatitis (1st for physicians, 2nd for pharmacists), fatigue/malaise (2nd for patients, 3rd for physicians), and rash/pruritus (1st for patients, 4th for pharmacists), though the degree of concern varied among the groups (Figure 2).

### 3.4. Reasons for Wanting Pharmacist Involvement in RCC Treatment Management

**Figure 3:**
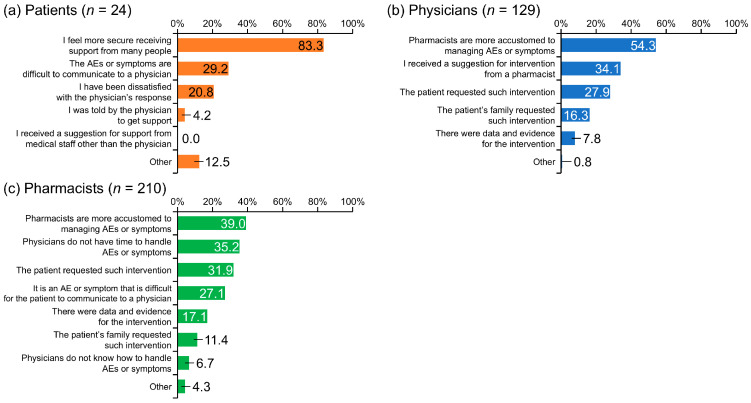
Reasons patients with renal cell carcinoma, physicians, and pharmacists felt that pharmacist involvement was needed. AE, adverse event.

The top three reasons patients gave for wanting pharmacist involvement were: “I feel more secure receiving support from many people” (83.3%), “The AEs or symptoms are difficult to communicate to a physician” (29.2%), and “I have been dissatisfied with the physician’s response” (20.8%) (Figure 3a).

Physicians gave the following top three reasons for wanting pharmacist intervention: “Pharmacists are more accustomed to managing AEs or symptoms” (54.3%), “I received a suggestion for intervention from a pharmacist” (34.1%), and “The patient requested such intervention” (27.9%) (Figure 3b).

The main reasons given by pharmacists for the value of pharmacist involvement were: “Pharmacists are more accustomed to managing AEs or symptoms” (39.0%), “Physicians do not have time to handle AEs or symptoms” (35.2%), “The patient requested such intervention” (31.9%), and “It is an AE or symptom that is difficult for the patient to communicate to a physician” (27.1%) (Figure 3c).

### 3.5. Proposed Strategies to Strengthen Pharmacist Involvement in AE Management

**Figure 4:**
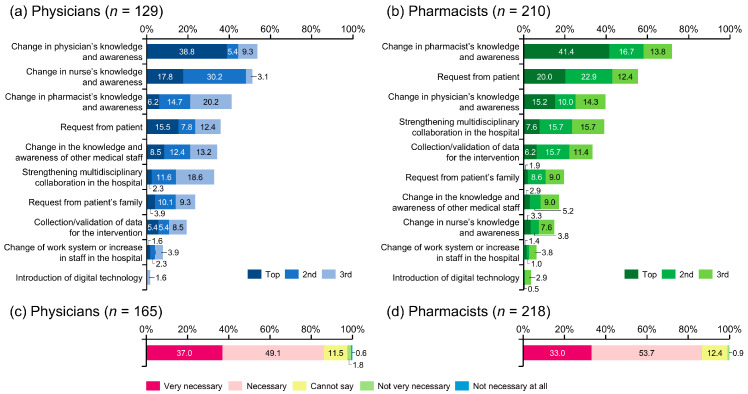
Measures deemed necessary to enhance pharmacist involvement in the management of adverse events. Regarding support from medical staff other than physicians, only answers from pharmacists were extracted for analysis.

From the perspective of physicians, the factors required to encourage pharmacist intervention in managing AEs were a change in the physician’s (53.5%), nurse’s (51.1%), and/or pharmacist’s (41.1%) knowledge and awareness; and/or a request from the patient (35.7%) (Figure 4a).

Pharmacists believed that the key to encouraging their involvement in the management of AEs was increasing their knowledge and awareness (71.9%), followed by patient requests (55.3%), physician’s knowledge and awareness (39.5%), and strengthening multidisciplinary collaboration in the hospital (39.0%) (Figure 4b).

Regarding the hospital-based cooperation system for pharmacists, 26.7% of physicians and 24.8% of pharmacists indicated that pharmaceutical outpatient clinics had been established at their facilities. These were considered either very necessary or necessary by 86.1% and 86.7% of physicians and pharmacists, respectively (Figure 4c,d).

## 4. Discussion

This study identified key aspects of pharmacist intervention in RCC treatment, including the percentages of patients, physicians, and pharmacists who consider such intervention to be necessary; the AEs for which pharmacist intervention is deemed essential; the reasons for supporting pharmacist involvement; and factors that may promote such interventions. There was significant variability in how patients and healthcare professionals perceived the necessity of pharmacist intervention. Some results suggested that among patients, there is insufficient understanding of the potential benefits of pharmacist intervention, such as having an alternative healthcare professional to consult regarding AEs that they find difficult to communicate with their physician about. These findings highlight the potential value of increasing patient awareness about the benefits of pharmacist involvement and support in patient care.

The degree to which pharmacists thought their involvement in managing AEs was necessary differed between patients and healthcare professionals, with 28.9% of patients, 78.2% of physicians, and 96.3% of pharmacists considering it necessary. Pharmacists have a diverse scope of practice [8], and the role and responsibilities of pharmacists in AE management differ substantially across healthcare systems, reflecting differences in pharmacist knowledge and/or the availability of medicines [17,18,19]. In Japan, the roles expected of pharmacists by non-medical personnel are fewer than the roles that pharmacists themselves recognize [20].

These results show that the degree to which patients and healthcare professionals think pharmacist involvement in managing treatment is necessary varies greatly. Furthermore, while 71.1% of patients indicated they did not feel the need for a pharmacist’s involvement in their treatment, 35.6% of these patients experienced AEs that were difficult to communicate to their physicians. It is possible that this indicates patients do not understand that the role of pharmacists includes patient counseling. It also suggests that there are AEs that can be improved through consultation with pharmacists. Therefore, when considering the role of pharmacists and the significance of their interventions, one issue that arises is the gap in the perception of the contributions pharmacists can make. Through patient education, better understanding of the role pharmacists may play in managing treatment may lead to improved communication between patients and healthcare professionals, and enhanced treatment [21].

The AEs and symptoms that required pharmacist intervention differed among patients, physicians, and pharmacists. The AEs that pharmacists considered requiring their involvement were mainly those for which supportive care has been established and can be addressed through pharmaceutical intervention, such as constipation [22], stomatitis [23], diarrhea [24], and nausea/vomiting [25]. In contrast, patients listed symptoms that interfered with their daily activities, such as rash/pruritus, fatigue/malaise, diarrhea, hand or foot pain, and stomatitis.

In addition to stomatitis, physicians listed symptoms that were difficult to improve through pharmacological intervention, such as anorexia [26], fatigue/malaise [27], dysgeusia [28], and depression [29]. These symptoms are often underreported and challenging for physicians to manage during busy outpatient visits; therefore, it is important that pharmacists actively participate in pharmaceutical interventions and AE monitoring for these symptoms. Further, patients in need of support could be referred to physicians, dietitians or mental health professionals by a pharmacist [30].

In a Japanese study, cancer patients who received support from pharmacists at outpatient clinics experienced a lower degree of malaise than those without such interventions [31]. Similarly, the incidence rates of grade ≥2 anorexia were significantly lower in patients with RCC receiving pazopanib because of comprehensive pharmaceutical intervention [32]. These studies suggest that pharmacists’ careful monitoring of various AEs may help prevent the occurrence and/or worsening of AEs. This may be because interventions such as pharmacist counseling are adequate for the early detection and management of AEs, including fatigue/malaise and anorexia. Furthermore, pharmacists could consult with physicians and dietitians regarding nutritional aspects or refer patients to them as necessary. The importance of this role is not limited to patients taking oral anticancer drugs but is equally essential for patients receiving ICIs [33]. Notably, in this study, the most common reason patients gave for wanting pharmacist intervention was that they feel more secure receiving support from many people.

There have been reports that cancer patients who receive consultation from pharmacists at pharmaceutical outpatient clinics, in addition to consultations with physicians, have a better understanding of drug therapy and management of AEs and are more satisfied with their treatment [34]. This suggests that pharmacist intervention may contribute to the early detection and improvement of AEs and improve patient satisfaction with treatment. The present study asked physicians and pharmacists why they felt pharmacist intervention was necessary. The most common response given by physicians and pharmacists was, “Pharmacists are more accustomed to handling the AE or symptom,” but 27.9% of physicians and 31.9% of pharmacists also responded that the patient requested such intervention.

Furthermore, 35.7% of physicians and 55.3% of pharmacists responded that patient requests would promote pharmacist intervention. These results show that for patients to receive appropriate pharmacist intervention and better treatment, it is necessary for patients themselves to be proactive in voicing their opinions to healthcare professionals. Consequently, patients who collaborated in decision-making were more satisfied with communication with healthcare professionals than those who were passive [35]. This suggests that patients’ active collaboration may lead to improved satisfaction with pharmacotherapy.

Establishing and strengthening a collaborative system within hospitals is also necessary to promote pharmacist involvement in cancer care. To encourage multidisciplinary collaboration in Japanese hospitals, a revised medical fee was introduced in April 2024. Under this new system, if pharmacists collect and assess patient information before the physician’s consultation (thereby enabling the physician to formulate a more appropriate treatment plan), the hospital can receive additional medical fee points. It is thought that this will encourage the establishment of pharmaceutical outpatient clinics. In addition to the reduction in the incidence of AEs associated with quality of life in cancer patients [36], various reports showed positive effects of pharmaceutical outpatient clinics, including a reduction in the working hours of physicians and an increase in hospital revenue due to an increase in the number of outpatients [37].

According to the present study results, only around 25% of hospitals established pharmaceutical outpatient clinics, but more than 85% of surveyed physicians and pharmacists responded that they thought pharmaceutical outpatient clinics were necessary. A previous systematic review revealed that pharmaceutical outpatient clinics have an impact on medication-related outcomes in patients receiving anticancer therapies across the world [38]. Therefore, it is hoped that pharmaceutical outpatient clinics will be used more in the future and that the benefits of this will be passed on to patients and healthcare professionals.

This study has some limitations. The questionnaire used is not validated, and the survey was conducted only in Japan, so it may not be generalizable to other populations. The number of patients with mRCC was limited, and the mean age of the patients was relatively lower than that of patients with mRCC in Japan in a previous report (44.2 vs. 66 years) [39]. Younger patients may be more likely to complete online surveys, resulting in a study population with younger participants, as we noted in a previous report [3]. Thus, further studies on this subject should be conducted in larger and older patient populations. Additionally, as the physicians and pharmacists surveyed were not necessarily those treating the RCC patients who participated in the survey, there is a possibility that this increased any gaps in the responses of patients and medical staff. Moreover, pharmacists manage medications across a wide range of areas rather than focusing on a single specialty, and while 49.1% of the pharmacists who responded to the survey had specific involvement in treating patients with RCC in the previous year, the majority did not. As this was an ad hoc analysis of a cross-sectional study, more robust studies, such as longitudinal or interventional studies, are needed to assess the real impact of pharmacist participation on AE management and patient satisfaction. Finally, we did not investigate how treatment line or treatment duration could affect the survey results.

## 5. Conclusions

There are differences among patients with RCC, physicians, and pharmacists regarding their expectations of pharmacist involvement and AEs they consider require intervention. Thus, it is important to raise awareness among patients and healthcare professionals about the role of pharmacists in systemic cancer therapy. Establishing pharmaceutical outpatient clinics alongside general outpatient clinics is expected to strengthen the collaborative system and improve treatment satisfaction among cancer patients. Therefore, it is necessary to further evaluate the effectiveness of pharmacist intervention in actual clinical practice, provide patient education, and foster stronger partnerships among healthcare professionals.

## Figures and Tables

**Table 1:** Background of participants.

Characteristic	Patients (*n* = 83)	Physicians (*n* = 165)	Pharmacists (*n* = 218)
Age (mean ± SD), years	44.2 ± 12.2		
Career as physician or pharmacist (mean ± SD), years		18.1 ± 7.6	18.5 ± 8.4
Sex, n (%)			
Male	53 (63.9)	152 (92.1)	151 (69.3)
Female	30 (36.1)	12 (7.3)	66 (30.3)
Other	0 (0.0)	1 (0.6)	1 (0.5)
Treatment history, n (%)			
Systemic therapy	83 (100)		
Ongoing	77 (92.8)		
Discontinued or interrupted	6 (7.2)		
Surgery	31 (37.3)		
Radiation therapy	3 (3.6)		
Duration of systemic therapy, n (%)			
<6 months	25 (30.1)		
6 months to 2 years	29 (34.9)		
>2 years	29 (34.9)		
Main type of facility they were working in, n (%)			
University hospital		35 (21.2)	22 (10.1)
Public hospital		53 (32.1)	37 (17.0)
General hospital		72 (43.6)	65 (29.8)
Clinic		5 (3.0)	2 (0.9)
Pharmacy/drugstore		0 (0.0)	92 (42.2)
Specialty, n (%)			
Urology		103 (62.4)	
Medical oncology		50 (30.3)	
Renal transplant surgery		12 (7.3)	
Experience in RCC treatment (mean ± SD), years		15.8 ± 7.4	
Experience in any cancer treatment (mean ± SD), years			9.0 ± 5.8

RCC, renal cell carcinoma; SD, standard deviation. “Public hospital” includes cancer centers, base hospitals for collaborative cancer care, and tertiary referral hospitals. “General hospital” refers to community hospitals other than public hospitals.

## Data Availability

The datasets generated and analyzed during the current study are available from the corresponding author upon reasonable request.

## Supplementary Materials

The following supporting information can be downloaded at: https://www.mdpi.com/article/10.3390/cancers17243951/s1, Figure S1: Patient disposition; Table S1: Web survey questionnaire.

## Abbreviations

The following abbreviations are used in this manuscript:

AE	Adverse event
ICI	Immune checkpoint inhibitor
mRCC	Metastatic RCC
RCC	Renal cell carcinoma
SD	Standard deviation
