# Supplementary material for: Perceptions and Expectations of Pharmacist Interventions in Adverse Event Management During Drug Therapy for Metastatic Renal Cell Carcinoma: A Cross-Sectional Survey in Japan
Source: Cancers (Basel). 2025 Dec 11;17(24):3951. doi: 10.3390/cancers17243951 (PMC12731063; doi:10.3390/cancers17243951)

Supplementary Table S1 Web survey questionnaire

**Patient**	
Q25	Of the AEs and symptoms from drug therapy for renal cell carcinoma that you have experienced up to this time, are there any for which you feel support from pharmacist is necessary?
	(Select all that apply to AEs and symptoms, respectively)
Q26	Please tell us the reason(s) that you felt the need for support from pharmacist for the following AEs and symptoms.
1	The AEs or symptoms are difficult to communicate to a physician.
2	I have been dissatisfied with the physician's response.
3	I feel more secure receiving support from many people.
4	I was told by the physician to get support.
5	I received a suggestion for support from medical staff other than the physician.
6	Other
**Physician**	
Q25	Of the AEs and symptoms from drug therapy for renal cell carcinoma, are there any for which you feel interventions by pharmacist are necessary? (Select all that apply)
Q26	Please tell us the reason(s) that you felt the need for intervention by pharmacist for the following AEs and symptoms.
1	Pharmacists are more accustomed to managing AEs or symptoms.
2	I received a suggestion for intervention from a pharmacist.
3	The patient requested such intervention.
4	The patient's family requested such intervention.
5	There were data and evidence for the intervention.
6	Other
Q27	Please tell us the top three things, in order, that are needed for interventions by pharmacist to proceed smoothly for the following AEs and symptoms.
1	Change in physician's knowledge and awareness
2	Change in nurse's knowledge and awareness
3	Change in pharmacist's knowledge and awareness
4	Change in the knowledge and awareness of other medical staff
5	Request from patient
6	Request from the patient's family
7	Collection/validation of data for the intervention
8	Strengthening multidisciplinary collaboration in the hospital
9	Change of work system or increase in staff in the hospital
10	Introduction of digital technology

Q35 Does the institution where you currently work have the following type(s) of specialty outpatient clinics or consulting services (handled by medical staff other than physicians) ? (Select all that apply)

1 Outpatient chemotherapy room
2 Pharmacist outpatient service
3 Nurse outpatient service
4 Consultation service (handled by medical staff other than physicians)
5 Other
6 None of them

Q37 To what degree do you think outpatient services by medical staff other than physicians are necessary?

1 Very necessary
2 Necessary
3 Cannot say
4 Not very necessary
5 Not necessary at all

Pharmacist

Q26 Of the AEs and symptoms from drug therapy for renal cell carcinoma, are there any for which you feel interventions by a pharmacist are necessary? (Select all that apply)

Q27 Please tell us the reason(s) that you felt the need for intervention by a pharmacist for the following AEs and symptoms.

1 It is an AE or symptom that is difficult for the patient to communicate to a physician.
2 Physicians do not know how to handle AEs or symptoms.
3 Physicians do not have time to handle AEs or symptoms.
4 Pharmacists are more accustomed to handling AEs or symptoms.
5 The patient requested such intervention.
6 The patient’s family requested such intervention.
7 There were data and evidence for the intervention.
8 Other

Q28 Please tell us the top three things, in order, that are needed for intervention by a pharmacist to proceed smoothly for the following AEs and symptoms.

1 Change in physician’s knowledge and awareness
2 Change in nurse’s knowledge and awareness
3 Change in pharmacist’s knowledge and awareness
4 Change in the knowledge and awareness of other medical staff
5 Request from patient
6 Request from patient’s family
7 Collection/validation of data for the intervention
8 Strengthening multidisciplinary collaboration in the hospital
9 Change of work system or increase in staff in the hospital
10 Introduction of digital technology

11	Nothing in particular
12	Do not know
Q36	Does the institution where you currently work have the following type(s) of specialty outpatient clinics or consulting services (handled by medical staff other than physicians) ? (Select all that apply)
1	Outpatient chemotherapy room
2	Pharmacist outpatient service
3	Nurse outpatient service
4	Consultation service (handled by medical staff other than physicians)
5	Other
6	None of them
Q38	To what degree do you think outpatient services by medical staff other than physicians are necessary?
1	Very necessary
2	Necessary
3	Cannot say
4	Not very necessary
5	Not necessary at all

AE, adverse event.

Supplementary Figure S1 Patient disposition

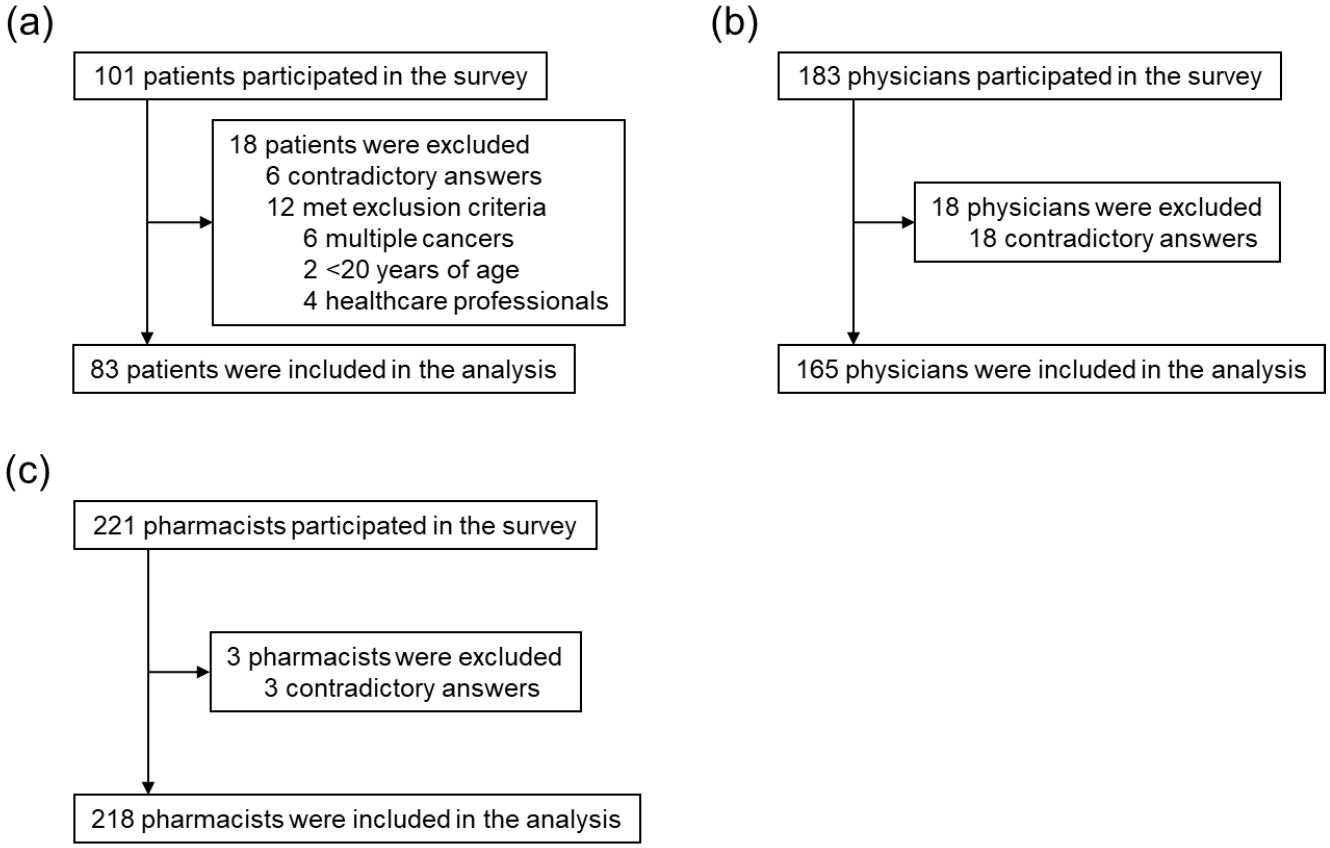

Supplement: Supplementary file 1 [file cancers-17-03951-s001.zip › cancers-3956090-supplementary.pdf]
